# Comparison of cervical cancer screening among women with and without hysterectomies: a nationwide population-based study in Korea

**DOI:** 10.1186/s12885-018-4723-9

**Published:** 2018-08-11

**Authors:** Ji-Yeon Shin, Kui Son Choi, Mina Suh, Boyoung Park, Jae Kwan Jun

**Affiliations:** 1 Department of Preventive Medicine, School of Medicine, Kyungpook National University, Daegu, 41944 Republic of Korea; 2 Graduate School of Cancer Science and Policy, National Cancer Center, 323 Ilsan-ro, Ilsandong-gu, Goyang, 10408 Republic of Korea; 3 National Cancer Control Institute, National Cancer Center, 323 Ilsan-ro, Ilsandong-gu, Goyang, 10408 Republic of Korea; 4 Department of Medicine, College of Medicine, Hanyang University, Seoul, 04763 Republic of Korea

**Keywords:** Uterine cervical neoplasms, Mass screening, Hysterectomy, Papanicolaou test, Guideline

## Abstract

**Background:**

Cervical cancer screening is not recommended for women who underwent hysterectomy with no history of cervical intraepithelial neoplasia (CIN) of grade 2 or higher. We aimed to determine the cervical cancer screening rate in Korean women who underwent hysterectomies and compare it to that in women with intact uteri.

**Methods:**

We used data from the 2014–2016 Korean National Cancer Screening Survey; 6807 women aged 30–74 years were included in the study. Participants were asked about their experiences with cervical cancer screening, hysterectomy status, and other variables associated with cancer screening.

**Results:**

The screening rates among women who have undergone a hysterectomy vs. those who have not during the past 2 years were 61.8% (95% confidence interval [CI], 58.8–64.9) and 64.7% (95% CI, 64.1–65.3), respectively. Among younger women (30–44 years) and women with a family history of cancer, those with hysterectomies showed a higher cervical cancer screening rate than those without (77.8% vs. 57.1% and 75.0% vs. 67.1%, respectively).

**Conclusions:**

Despite available evidence and clinical recommendations, a considerable number of Korean women who no longer have a cervix continue to undergo unnecessary cervical cancer screening. It is necessary to identify the exact underlying causes for this phenomenon, and systematic efforts are required to prevent unnecessary screening for women who have undergone a hysterectomy.

**Electronic supplementary material:**

The online version of this article (10.1186/s12885-018-4723-9) contains supplementary material, which is available to authorized users.

## Background

Cervical cancer is one of the most common cancers among women worldwide. Approximately 530,000 new cases of cervical cancer were diagnosed in 2012, and 270,000 women died of the disease [1].Therefore, several countries have made efforts to reduce the burden of this cancer, and one such endeavor is promoting cervical cancer screening programs [2, 3]. Since the introduction of cervical cancer screening in the 1950s and 1960s, the incidence and mortality rates due to cervical cancer have dramatically decreased in western countries [4–6]. In Korea, screening programs were formulated by the Korean government in 1996; the government formally established the National Cancer Screening Program (NCSP) for cervical cancer in 1999. The NCSP recommends that women aged ≥30 years undergo cervical cancer screening on a biennial basis via the Papanicolaou (Pap) smear [7]. Since then, the age-standardized incidence rate of cervical cancer has been decreasing steadily from 16.3 per 100,000 in 1995 to 9.0 per 100,000 in 2014 [8].

Hysterectomy is the most common non-pregnancy related surgical procedure for women of reproductive age [9, 10]. More than one-third of all women in the United States have had a hysterectomy by the age of 60 years [11]. In Korea, hysterectomies were the sixth most common type of surgery in 2015 [12]. According to recent consensus guidelines, cervical cancer screening is not recommended for women who undergo total hysterectomy for benign diseases [6, 13–15]. In 1996, the United States Preventive Services Task Force (USPSTF) first recommended that women discontinue screening if they have undergone a total hysterectomy and had no history of cervical cancer [6, 13]. In 2012, the American Cancer Society and the American College of Obstetricians and Gynecologists recommended that women of all ages should forgo screening for vaginal cancer using any modality following a hysterectomy that included removal of the cervix if they have no history of cervical intraepithelial neoplasia (CIN) of grade 2 or higher [15]. In Korea, the National Cervical Cancer Screening Guideline Development Committee stated in 2015 that cervical cancer screening is not recommended for women who underwent a total hysterectomy [16].

Despite the 1996 USPSTF recommendations, the 2010 National Health Interview Survey found that 55.8% of women in the United States who had hysterectomies still reported having undergone cervical cancer screening in the prior three years [17]; the USPSTF also pointed out the unnecessary use of healthcare resources and ensuing costs resulting from this phenomenon. However, no studies have examined the actual status of cervical cancer screening for women who received hysterectomies in Korea. In this study, we aimed 1) to review and summarize the cervical cancer screening recommendations for women who underwent total hysterectomies and 2) to determine and compare the rate of cervical cancer screening among women with and without hysterectomies in Korea.

## Methods

### Study design and population

We used data from the Korean National Cancer Screening Survey (KNCSS) performed between 2014 and 2016. The KNCSS is a continuous nationwide cross-sectional interview survey conducted by the Korean National Cancer Center annually since 2004 to examine the participation rates of Koreans in screening for five common cancers: gastric, liver, colorectal, breast, and cervical. The details of the surveying methods were described previously [7]. Briefly, survey participants were selected based on the Resident Registration Population data using a stratified, multistage, random sampling procedure according to residential area, age, and sex. Investigators from a professional research agency conducted face-to-face interviews in the participants’ homes. They obtained informed consent from all participants. Eligible participants were asked about their experiences related to screening for the five aforementioned cancers and provided information on health behaviors, health status, family history of cancer, and sociodemographic factors.

Surveys were completed by 4500 participants aged 20 years or older in 2014, 4500 in 2015, and 4500 in 2016; the response rates were 40.9, 66.0 and 48.1%, respectively. We selected 6807 cancer-free women aged 30–74 years as the study population, as women aged ≥30 years are eligible to undergo cervical cancer screening biennially via Pap smear, according to the NCSP protocols [7], and cervical cancer screening is recommended to be terminated at the age of 74 years, after three consecutive negative cervical cancer screening results [16]. Since 2005, the NCSP has provided Medical Aid recipients and NHI beneficiaries in the lower 50% income stratum with cervical cancer screening free of charge. NHI beneficiaries in the upper 50% income stratum receive cervical cancer screening services from the NHI Corporation and pay 10% of the screening cost [7]. All participants provided answers to the relevant questions regarding experience or timing of cervical cancer screening as well as undergoing hysterectomy and any Pap smears beforehand.

### Measures

Using a structured self-reported questionnaire (Additional file 1), participants were probed regarding their experiences with cervical cancer screening and hysterectomy status. In terms of hysterectomy, participants were asked: “Have you received a hysterectomy?” and “When did you received a hysterectomy?” Regarding cervical cancer screening, participants were asked “Have you ever undergone cervical cancer screening?” and “When was the last time you had a cervical cancer examination?” The ‘cervical cancer screening rate with recommendation’ was defined as the proportion of participants who underwent a Pap smear within the previous two years according to NCSP recommendations.

As in previous studies investigating factors associated with cancer screening [18, 19], we considered age group (30–44, 45–64, and 65–74 years), residential area (metropolitan, urban, or rural), education (≤11 or ≥ 12 years of schooling), family history of cancer (yes or no), health insurance status (medical aid program [MAP] or the National Health Insurance [NHI]), possession of private health insurance (yes or no), and monthly household income group (< 2500, 2500–4499, or ≥ 4500 USD) as covariates.

### Statistical analysis

We used the chi-square test to investigate the relationships between sociodemographic characteristics among women with vs. without hysterectomies. Univariable and multivariable logistic regression models were used to compare the cervical cancer screening rates between these two groups within each subgroup of covariates. In the multivariable analysis, all covariates except the subgroup variable were included in the model, and the adjusted odds ratios (aORs) and 95% confidence intervals (CIs) were calculated. For example, in order to calculate the aOR of undergoing cervical cancer screening in ages 30–44 years, a logistic regression analysis was performed for ages 30–44 years that included hysterectomy status (reference group: reporting no hysterectomy), and other covariates, except for age-groups, as independent variables. All statistical analyses were performed using the SAS software package (version 9.4, SAS Institute, Cary, NC, USA), and appropriate sampling weights were included with a survey procedure to reflect the complex survey design. Sampling weight is a measure of the number of people in the population and represents a sample of the KNCSS and reflects the unequal probability of selection, nonresponse adjustment, and adjustment to independent population controls. The use of sample weights allows us to obtain an unbiased national estimate. This study was approved by the Institutional Review Board of the National Cancer Center, Korea (approval no. NCCNCS-08-129).

## Results

Table 1 summarizes the cervical cancer screening guidelines for women who have undergone a hysterectomy. In 1996, the USPSTF first recommended against cervical cancer screening for women who have undergone a total hysterectomy for benign disease [13]; two subsequent cervical cancer screening guideline updates by the USPSTF in 2003 and 2012 [6, 20] maintained the same guidelines. The USPSTF recommended against screening for cervical cancer in women who have had a hysterectomy with removal of the cervix and who do not have a history of a high-grade precancerous lesion (CIN grade 2 or 3) or cervical cancer (D recommendation) in 2012 [6]. In Korea, the National Cervical Cancer Screening Guideline Development Committee published in 2015 stated that cervical cancer screening is not recommended for women who underwent total hysterectomy and had no history of CIN grade 2 or higher lesions [16].

**Table 1:** Cervical cancer screening guidelines for women who have undergone a hysterectomy

Guideline	Year	Recommendation	Ref.
ASCCP/ACS/ASCP	2012	Women at any age following a hysterectomy with removal of the cervix who have no history of CIN2+ should not be screened for vaginal cancer using any modality.	[15]
USPSTF	2012	There is convincing evidence that continued screening after hysterectomy with removal of the cervix for indications other than a high-grade precancerous lesion or cervical cancer provides no benefits.	[6]
NCCN	2010	Women who have had a total hysterectomy for benign indications and have no prior history of high-grade CIN should discontinue routine cytology testing.	[14]
KSGO	2012	Women who have undergone hysterectomy should continue with screening tests if they have a history of CIN grade 2 or higher or if they do not know their pervious cytology results.	[30]
NCSP	2015	Cervical cancer screening was not recommended for women who underwent hysterectomy and women with no history of CIN grade 2 or higher.	[16]

*ASCCP* American Society for Colposcopy and Cervical Pathology, *ACS* American Cancer Society, *ASCP* American Society for Clinical Pathology, *USPSTF* U.S Preventive Services Task Force, *NCCN* National Comprehensive Cancer Network, *KSGO* Korean Society of Gynecologic Oncology, *NCSP* National Cancer Screening Program, *CIN* Cervical Intraepithelial Neoplasia

Of the 6807 survey respondents, 249 (3.7%) reported having undergone a hysterectomy. Table 2 shows the general characteristics of women who underwent a hysterectomy vs. those who did not. Women who underwent hysterectomies were older, less educated, and had lower incomes than women who did not undergo hysterectomies. The proportions of women who underwent Pap smears in the previous year were 30.9% in the hysterectomy group and 30.3% in the non-hysterectomy group, indicating similar proportions.

**Table 2:** General characteristics of the study population

Characteristics	Reporting no hysterectomy		Reporting hysterectomy		*p*-value
	n	Weighted %	n	Weighted %	
Age, years					
30–44	2436	37.2	18	7.2	< 0.001
45–64	3392	51.7	167	67.1	
65–74	730	11.1	64	25.7	
Residence area					
Metropolitan	3003	45.8	122	49.0	0.335
Urban	3036	46.3	113	45.4	
Rural	519	7.9	14	5.6	
Education, years					
≤ 11	897	13.7	68	27.3	< 0.001
≥ 12	5661	86.3	181	72.7	
Family history of cancer					
Yes	887	13.5	80	32.1	< 0.001
No	5671	86.5	169	67.9	
Health insurances status					
NHI	6480	98.8	236	94.8	< 0.001
MAP	78	1.2	13	5.2	
Private health insurance					
Yes	5628	85.8	199	79.9	0.009
No	930	14.2	50	20.1	
Monthly household income, USD^a^					
< 2500	1059	16.2	56	22.5	0.013
2500–4499	3199	48.8	103	41.4	
≥ 4500	2300	35.1	90	36.1	
Time since most recent Pap smear test, years					
< 1	1985	30.3	77	30.9	0.003
1–2	2260	34.5	77	30.9	
> 2	628	9.6	41	16.5	
Never	1685	25.7	54	21.7	

*NHI* National Health Insurance, *MAP* Medical Aid Program

^a^1 USD = 1000 KWN

The screening rates among women who underwent a hysterectomy during the previous two years vs. those who did not were 61.8% (95% CI, 58.8–64.9) and 64.7% (95% CI, 64.1–65.3), respectively (Table 3). In the hysterectomy group, a younger age and higher income level coincided with a higher screening rate. Among participants who were 30–44 years of age or participants with a family history of cancer, those who underwent hysterectomies had higher screening rates than those who did not (77.8% vs. 57.1% and 75.0% vs. 67.1%, respectively).

**Table 3:** Cervical cancer screening rates according to hysterectomy status and sociodemographic variables

Characteristics	Reporting no hysterectomy		Reporting hysterectomy		Reporting hysterectomy vs. reporting no hysterectomy			
	Screening rate	(95% CI)	Screening rate	(95% CI)	cOR	(95% CI)	aOR	(95% CI)
Overall	64.7	(64.1–65.3)	61.8	(58.8–64.9)	0.87	(0.67–1.13)	0.75	(0.57–0.98)
Age, years								
30–44	57.1	(56.1–58.1)	77.8	(68.0–87.6)	2.70	(0.88–8.22)	2.36	(0.75–7.44)
45–64	70.1	(69.3–70.9)	64.7	(61.0–68.4)	0.77	(0.56–1.07)	0.75	(0.54–1.05)
65–74	64.9	(63.2–66.7)	50.0	(43.8–56.3)	0.55	(0.33–0.93)	0.53	(0.31–0.91)
Residence area								
Metropolitan	65.8	(64.9–66.7)	58.2	(53.7–62.7)	0.72	(0.50–1.04)	0.59	(0.40–0.88)
Urban	64.1	(63.3–65.0)	66.4	(61.9–70.8)	1.07	(0.72–1.60)	0.97	(0.65–1.46)
Rural	62.0	(59.9–64.2)	57.1	(43.9–70.4)	0.83	(0.28–2.44)	0.75	(0.24–2.31)
Education, years								
≤ 11	67.2	(65.7–68.8)	64.7	(58.9–70.5)	0.82	(0.49–1.39)	0.92	(0.54–1.56)
≥ 12	64.3	(63.7–65.0)	60.8	(57.1–64.4)	0.87	(0.64–1.18)	0.69	(0.50–0.95)
Family history of cancer								
Yes	67.1	(65.5–68.7)	75.0	(70.2–79.8)	1.47	(0.87–2.49)	1.42	(0.82–2.44)
No	64.4	(63.7–65.0)	55.6	(51.8–59.4)	0.69	(0.50–0.94)	0.59	(0.43–0.81)
Health insurance status								
NHI	64.8	(64.2–65.4)	62.7	(59.6–65.9)	0.90	(0.69–1.18)	0.75	(0.57–0.99)
MAP	57.7	(52.1–63.3)	46.2	(32.3–60.0)	0.64	(0.19–2.15)	0.86	(0.19–3.90)
Private health insurance								
Yes	66.0	(65.4–66.7)	62.8	(59.4–66.2)	0.88	(0.65–1.17)	0.70	(0.52–0.95)
No	56.9	(55.3–58.5)	58.0	(51.0–65.0)	0.96	(0.54–1.73)	1.01	(0.55–1.85)
Monthly household income, USD^a^								
< 2500	65.3	(63.9–66.8)	48.2	(41.5–54.9)	0.48	(0.28–0.82)	0.49	(0.28–0.86)
2500–4499	61.9	(61.0–62.7)	63.1	(58.4–67.9)	1.03	(0.69–1.55)	0.86	(0.57–1.31)
≥ 4500	68.4	(67.5–69.4)	68.9	(64.0–73.8)	1.05	(0.66–1.65)	0.87	(0.54–1.41)

*CI* confidence intervals, *cOR* crude odds ratio, *aOR* adjusted odds ratio, Adjustments were made for age, residence area, education, family history of cancer, health insurance status, private health insurance, and monthly household income, *NHI* National Health Insurance, *MAP* Medical Aid Program

^a^1 USD = 1000 KWN

Table 3 shows the results of multivariable analyses performed to identify factors associated with cervical cancer screening in the hysterectomy group vs. the non-hysterectomy group. Women who underwent hysterectomies in the following categories were less likely to undergo cervical cancer screening than their counterparts with intact uteri: Metropolitan residents, those with longer education, those with no family history of cancer, those who used the NHI, those with private health insurance, and those with monthly household incomes group < 2500 USD. Even after controlling for the other variables, subjects in the 30–44-year age group and those with a family history of cancer were more likely to undergo cervical cancer screening than women who had not undergone hysterectomies, although the differences were not were significant.

## Discussion

We found that 61.8% of women with hysterectomies underwent cervical cancer screening within the previous 2 years, despite the consensus of recommendations that women discontinue Pap smear examinations if they undergo a total hysterectomy following a diagnosis with a benign disease. Among women who did not undergo hysterectomies, 64.7% reported that they had undergone cervical cancer screening within the same period.

There are some possible explanations for the unnecessary screening of women who underwent hysterectomies. First, such women are unlikely to recognize that cervical cancer screening is no longer necessary. Second, it is possible that doctors may not be aware that women without cervices no longer require cervical cancer screening. Third, even if the doctors are aware of the recommendations, it is possible that they did not advise their patients of the same. In the absence of a shared medical record system, it is difficult for doctors to determine whether a patient underwent a total or subtotal hysterectomy; hence, a physician may not wish to encourage a patient to forgo cancer screening without knowledge of whether the patient is at a high risk for cervical cancer. Fourth, various medical environments and systems may produce unnecessary screenings. For example, 1- some screening examination systems are not under the coordination of the primary care physician in the primary care setting, 2- the national medical checkup system is followed automatically without consulting a physician beforehand, and 3- the fee-for-service system drives physicians to see more patients for shorter durations; all these factors may help explain our findings.

Despite several decades having passed since the publication of recommendations for women who underwent hysterectomy in the United States, cervical cancer screening rates in that country remain high. In 1992, 68.5% of women with a history of hysterectomy reported having had cervical cancer screening within the preceding three years. In 2002, 69.1% had undergone screening during the previous three years, which was not significantly different than the 1992 rate despite the 1996 USPTF recommendation that routine cervical cancer screening is unnecessary for women who have undergone a complete hysterectomy for benign disease [21]. In 2010, 64.8% of women in the United States with a history of hysterectomy reported having undergone cervical cancer screening; among women with no hysterectomies, 80.7% reported having undergone screening. This showed that unnecessary cervical cancer screening persists in women who have undergone hysterectomies [17].

According to the multivariable analyses that compared Pap smear screening rates between women with vs. without hysterectomies, women with hysterectomies who were residents of metropolitan areas, those with higher educational levels, those with higher incomes, and those with National Health Insurance underwent fewer Pap smear screening tests than those without hysterectomies. This result implies that subjects who are more sensitive and literate to health information, or are more amenable to healthcare guidance, actually follow recommendations and undergo less screening. Poor literacy is known to be linked to low socio-economic circumstances to some extent [22], and previous studies have shown that women with higher levels of education are less likely to undergo screening tests after being informed that such cancer screening is unnecessary [23].

However, in younger women (30–44 years) or women with a family history of cancer, those who had undergone hysterectomies showed higher cervical cancer screening rates than those who had not. In a recent Korean study, women who strongly believed that cancer screening facilitates early detection were less likely to change their intentions to undergo screening for thyroid cancer after receiving information about overdiagnosis [23]. In another study on breast cancer screening in the United Kingdom, women eligible for cost-free breast cancer screening under a nationwide screening program were less likely to defer continued screening after receiving information on overdiagnosis than women not eligible for such screening because of younger age [24]. Such individuals are likely to have greater interests and concerns about cancer. Therefore, while they actively seek health information on one hand but ignore guidelines regarding the non-necessity of screening on the other, as such recommendations may increase their anxiety or cause mental discomfort [25]. Once formed, ideas on healthcare are not easily changed because of a single piece of additional information. Given the experiences in the United States, it is important to understand selectivity in receiving information and provide accurate information repetitively.

Our study has some limitations. First, data were collected using self-reported questionnaires; hence, recall bias may exist and the estimates should be interpreted with caution. The validity of self-reported cancer screening may be over-reported [26], especially in women [27]. Nevertheless, self-reported screening behavior generally is in good agreement with medical records [28, 29], and many publications rely on this. Second, over- or underestimation may occur when determining the unnecessary cervical cancer screening rates owing to the lack of detail on the types or reasons for hysterectomies in the KNCSS data. Especially, we could not distinguish between total and subtotal (supracervical) hysterectomies, which remove only the uterus and leave the cervix intact, among women who answered that they had undergone a hysterectomy. Women with subtotal hysterectomies would still be recommended for screening, and this could, to some extent, affect our results, i.e. high screening rates among hysterectomized women. However, Korea has reportedly shown a relatively low proportion of cervical conservation, of 6.8% among overall hysterectomies in 2008 [10], which means that the impact will be limited. In future studies, it will be necessary to determine cervical cancer screening rates according to specific hysterectomy types (total hysterectomy or subtotal hysterectomy) as well as the reasons for them (i.e., benign or malignant conditions). Third, we could not determine causal associations between cervical cancer screening and hysterectomy status and other associated factors because the KNCSS uses a cross-sectional design. Fourth, the percent who reported having a hysterectomy - 3.7%- was likely underestimated although all participants responded to the question of hysterectomy history. It is possible that this has affected the outcome of our study. Despite such limitations, our study ought to be worthwhile as it is the first to determine the rate of unnecessary Pap smear screening among women with hysterectomies in Korea using nationwide representative, population-based data.

## Conclusions

In conclusion, our investigation revealed that a considerable number of women without cervices in Korea undergo unnecessary cervical cancer screening contrary to evidence-based clinical recommendations. Performing unnecessary screening can cause various side effects including psychosocial stress, wasting of limited healthcare resources, and ultimately increased healthcare expenses. It is necessary to identify the exact underlying causes for pursuing unnecessary screening, and systematic efforts are required to reduce such screening for women who have undergone total hysterectomies.

## Additional file

Additional file 1: Questionnaire hysterectomy and Pap screening. (DOCX 18 kb)

## Abbreviations

CIN: Cervical intraepithelial neoplasia
KNCCS: Korean National Cancer Screening Survey
NCSP: National Cancer Screening Program
USPSTF: United States Preventive Services Task Force
