# Supplementary material for: Comparison of cervical cancer screening among women with and without hysterectomies: a nationwide population-based study in Korea
Source: BMC Cancer. 2018 Aug 11;18:810. doi: 10.1186/s12885-018-4723-9 (PMC6087535; doi:10.1186/s12885-018-4723-9)
Supplement: Supplementary file 1 — Questionnaire hysterectomy and Pap screening. (DOCX 18 kb) [file 12885_2018_4723_MOESM1_ESM.docx]

**Questionnaire about cervical cancer screening via Pap test in Korean women with or without hysterectomies**

1. Have you ever get screened with a Pap test?

① Yes (→ Q 1-1) ② No

1.1. When was the last experience of getting screening?

① Within 12 months ② 13-24 months ③ 25 months and over

2. Have you ever received a hysterectomy (surgery that removed the uterus)?

① Yes (→ Q 2-1) ② No

2-1. When did you have a hysterectomy?

Year □□□□

3. Are you covered by private supplemental insurance for cancer?

① Yes ② No

4. What is your final academic background?

① Uneducated ② Primary school ③ Middle school

④ High school ⑤ College ⑥ Above college

5. What is the total average monthly income for all family members in your household?

① No income

② Less than ￦1,000,000 KRW

③ ￦1,000,000 - ￦1,990,000 KRW

④ ￦2,000,000 - ￦2,990,000 KRW

⑤ ￦3,000,000 - ￦3,990,000 KRW

⑥ ￦4,000,000 - ￦4,990,000 KRW

⑦ ￦5,000,000 - ￦6,990,000 KRW

⑧ ￦7,000,000 - ￦9,990,000 KRW

⑨ More than ￦10,000,000 KRW

6. Is there anyone in your family (parents, brothers, sisters, and children) has cancer?

① Yes ② No
